# Towards a Transversal Definition of Psychological Resilience: A Literature Review

**DOI:** 10.3390/medicina55110745

**Published:** 2019-11-16

**Authors:** Antonella Sisto, Flavia Vicinanza, Laura Leondina Campanozzi, Giovanna Ricci, Daniela Tartaglini, Vittoradolfo Tambone

**Affiliations:** 1 Clinical Psychological Service, Campus Bio-Medico University Hospital, 00128 Rome, Italy; a.sisto@unicampus.it (A.S.); f.vicinanza@unicampus.it (F.V.); 2 Institute of Philosophy of Scientific and Technological Practice, Campus Bio-Medico University, 00128 Rome, Italy; v.tambone@unicampus.it; 3 School of Law, Medico-Legal Section, University of Camerino, 62032 Camerino (Macerata), Italy; giovanna.ricci@unicam.it; 4 Department of Professional Health Care Services, Campus Bio-Medico University Hospital, 00128 Rome, Italy; d.tartaglini@unicampus.it

**Keywords:** psychological, resilience, definition, perseverance, well-being

## Abstract

*Background and objectives*: This paper addresses psychological resilience, a multidisciplinary theoretical construct with important practical implications for health sciences. Although many definitions have been proposed in several contexts, an essential understanding of the concept is still lacking up to now. This negatively affects comparisons among research results and makes objective measurement difficult. The aim of this review is to identify shared elements in defining the construct of resilience across the literature examined in order to move toward a conceptual unification of the term. *Materials and methods*: A literature review was performed using the electronic databases ‘PubMed’ and ‘PsycINFO’. Scientific studies written in English between 2002 and May 2019 were included according to the following key terms: ‘Psychological’, ‘resilience’, and ‘definition’. *Results:* The review identifies five macro-categories that summarize what has been reported in the recent literature concerning the resilience phenomenon. They serve as a preliminary and necessary step toward a conceptual clarification of the construct. *Conclusions:* We propose a definition of psychological resilience as the ability to maintain the persistence of one’s orientation towards existential purposes. It constitutes a transversal attitude that can be understood as the ability to overcome the difficulties experienced in the different areas of one’s life with perseverance, as well as good awareness of oneself and one’s own internal coherence by activating a personal growth project. The conceptual clarification proposed will contribute to improving the accuracy of research on this topic by suggesting future paths of investigation aimed at deeply exploring the issues surrounding the promotion of resilience resources.

## 1. Introduction

### 1.1. Resilience: Historical and Cultural Development of the Concept

Over the years, much attention has been directed at the nature of resilience and how to best assess it. An extensive literature highlights the historical and cultural evolution of the concept, which assumed different shades of meaning over time. 

The early studies on resilience focused on understanding why only some individuals can react to adversity in a positive way by transforming them into opportunities for growth and new adaptation [1]. Living with a condition of adversity typically encompasses negative life circumstances that are known to be statistically associated with adjustment difficulties [2]. Furthermore, the concept of adversity can be identified as the state of suffering and discomfort aroused by a difficulty, misfortune, or potentially traumatic event [3]. After the Second World War, researchers began to investigate how people overcome traumatic events which can cause psychological distress. Issues concerning the possibility of transforming a destabilizing event into a personal search engine, the ability to integrate lights and shadows, resources and vulnerability, or suffering and courage started to become primary subjects of research aimed at providing a better understanding of the processes of resilience.

In particular, case studies of soldiers with post-traumatic stress disorder, as well as other forms of pathologies which have been diagnosed as results of traumatic events experienced in war, provided descriptions of individual characteristics of war veterans, highlighting at the same time that a significant number of subjects were able to effectively process the traumatic events experienced [4]. Later, research involving the analysis of risk and protective factors for mental health began to focus on the context of developmental psychology with the aim of exploring the different life trajectories of those subjects that had experienced trauma. This has led to the idea that resilience is much more than the ability to continue developing one’s skills despite adversity or to resist trauma by protecting oneself from the influence of external circumstances. It expresses the ability to react positively despite difficulties, turning them into opportunities for growth. Therefore, psychological resilience refers to a dynamic process that takes shape as a change allowing one to find a new balance and to evolve positively [2]. During this process of change, the individual develops new skills and a renewed feeling of personal efficacy and self-enhancement. This circular mechanism helps to implement the resilience process and its whole development. Thus, the change in an allostatic process is something necessary in order to adapt to changes produced by the environment [5].

In their resilience model, Richardson et al. [6,7] attempt to integrate two perspectives by considering them both as genetically determined traits and as processes. According to the authors, we all have an innate propensity for resilience, which can allow us to face difficulties and the breakdown of a pre-existing balance. The paradigm of destabilization of an individual’s life paradigm provides the opportunity for an in-depth self-reflection upon him- or herself and for a redefinition of the self. From the experience of insight and the search for one’s own inner resources comes the identification and reinforcement of the resiliency features. Consequently, the subject will enable strategies aimed at facing the adverse condition and rebuilding their own balance.

This model assumes the circularity of the influence of the self and the environment insofar as resilience is at the same time part of the adaptation process and of its outcome. Rutter, drawing upon studies on children born to schizophrenic mothers and showing that many of them had no abnormal behavior as adults [8], proposed an early definition of resilience as a ‘positive’ response of a subject to stress and adverse conditions. Here, ‘positive’ means the absence of psychopathological consequences (i.e., conduct disorders, affective, etc.). This study was preceded by a longitudinal study by Werner and Smith [9], which lasted 30 years and was conducted on a sample of 698 children born in Kauai (Hawaii). These subjects had been enrolled in the study because they had been exposed to different risk factors (difficult birth, poverty, families with problems with alcoholism, mental illness, aggression, etc.) that could have influenced their development towards the onset of mental illness. The survey allowed the authors to monitor the evolution of the sample’s emotional and relational adaptability over time. Despite the presence of multiple risk factors and the development of severe symptoms of psychopathological maladjustment in many of these children, the study showed that 28% of subjects achieved a good level of adaptation, becoming competent and self-confident adults with a satisfactory level of affective and social functionality. Building on these data, Werner defined resilience as the consolidation of the subject’s skills in adverse situations.

With Werner’s pioneering works, a fundamental change began to take place in research on resilience. It consisted of a shift from the analysis of risk and discomfort factors to the study of protective factors. Specifically, attempts were made to identify what characterizes resilient subjects and what factors enable the activation of positive processes when critical or emotionally painful life conditions are encountered. The results of these first investigations showed the presence of subjects who were defined as ‘resilient’ because they presented satisfactory or positive evolutionary results despite an unfavorable condition [10]. This evidence gave the concept of resilience an important visibility in the development of the salutogenic perspective [11] to the extent that it is used as a wide-ranging construct from a heuristic point of view for understanding normal health processes. In particular, Antonovsky argued that stress is an unavoidable phenomenon. However, a significant percentage of individuals can find their own balance and can grow and maintain a state of well-being despite adversities. For this reason, the author underlined the importance of orienting research towards the elements that allow this development and are at the origin of health (salutogenetic factors).

The debate continued between those who theorize resilience as a stable trait of personality [12,13] and those who define it as a dynamic process that varies in relation to contexts [14,15].

More recently, those who consider resilience in terms of a trait, in line with the ‘ego resiliency’ perspective [16], argue that personality characteristics are the main protective factors against stressors.

The authors who define resilience in terms of process [17,18] consider it a resource on which the success of the transaction between the individual and his context depends. According to this approach, protective and risk factors act simultaneously and dynamically, and the effect is the result of their interaction.

Therefore, being resilient means building and reconstructing one’s life path by restoring a new balance and producing a change in oneself [19].

### 1.2. The Complexity of the Resilience Phenomenon

The broad and articulated research carried out on resilience has highlighted the versatility of the construct and made the attempt to reach a shared definition of the concept more complex.

Over the years, many definitions of the term ‘resilience’ have been proposed to describe the construct. Although differing in their theoretical references and in the factors highlighted, they share a common vision of resilience as a complex phenomenon and the identification of numerous interacting variables. To date, the literature agrees that there are two necessary and adequate conditions for identifying the dynamics of the resilience process: Exposure to a significant risk and positive evolution in terms of psychosocial well-being despite the threat to which one is subjected [20]. A ‘significant risk’ refers to any element of a situation that is perceived as lacking a reachable solution and that can lead to a dysfunctional adaptation and to a condition of psychological distress [21,22]. In this perspective, resilient individuals would be able to rework their individual existence thanks to a ‘positive evolution’ of their life project despite the surrounding conditions. They develop the ability to integrate suffering and psychic vulnerability with personal, family, relational, and existential resources, managing to expand them according to their own needs. Though there seems to be a common ground in the work of research focusing on the process of psychological resilience, the concept is used in many ways depending in part on the area of application, with several implications from a theoretical and practical point of view. Consequently, these discrepancies hinder a shared definition of the construct and limit comparisons among research results, making objective measurement difficult. Thus, the importance of moving towards a conceptual unification of the term becomes evident.

### 1.3. Aims

Based on this premise, we engaged in a literature review of definitions of the term ‘psychological resilience’ aimed at identifying shared elements in defining the construct across the works examined. Findings were used for proposing a broad definition of psychological resilience that considers the various theoretical and multidisciplinary backgrounds associated with it and the different areas of application. This conceptual unification could be a useful starting point for future research focused on identifying effective training strategies to promote and support resilience resources and thus the personal well-being.

## 2. Materials and Methods

A literature review of the term ‘psychological resilience’ was performed by using the electronic databases ‘PubMed’ and ‘PsycINFO’. Scientific articles published between 2002 and May 2019 were reviewed according to the following key terms: ‘psychological’, ‘resilience’, and ‘definition’. The initial search in PubMed using the keywords ‘psychological’ and ‘resilience’ produced 7553 results. Subsequently an additional filter was added, the keyword ‘definition’, in order to focus exclusively on the articles that consider definitions of psychological resilience. This filter significantly reduced the number of publications to 82. These studies were further filtered by applying the following exclusion criteria.

Articles not written in English and those that did not contain a definition of the resilience after the full text screening were removed. In the end, 58 articles were selected through this process for the identification and analysis of the definitions, highlighting recurrent elements and specific characteristics.

**Figure 1:**
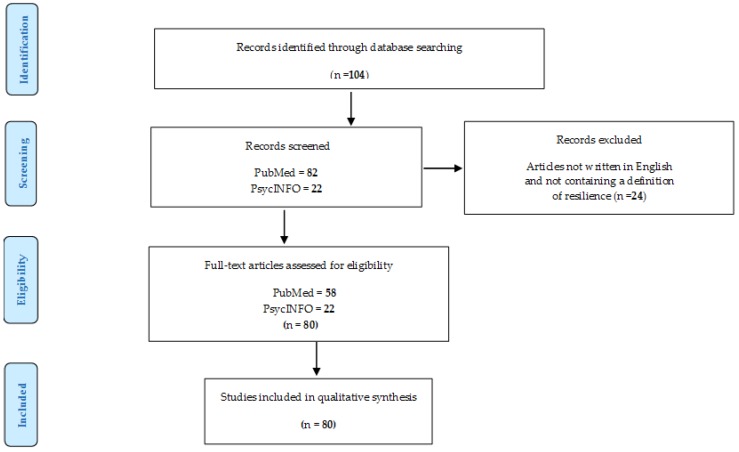
PRISMA checklist showing process of articles selection for inclusion in the literature review.

The same search with the same filters was repeated in the PsycINFO online database. As a result, 22 articles were singled out, as shown in Figure 1.

The search ultimately produced 126 definitions of psychological resilience developed by 109 work groups, and each of them has been catalogued by content and authors. Data were independently reviewed by two authors, and then compared and discussed to reach a consensus. After analyzing these results, five macro-categories were identified by taking into account the specific elements of each definition.

## 3. Results

The five macro-areas identified summarize what has been reported in the literature in recent years concerning description of the resilience phenomenon. They serve as a preliminary and necessary step to identifying the transversal elements that can comprise a broad definition of ‘psychological resilience’. Each macro-category focuses on a specific feature of the resilience construct, helping to highlight its complex nature and its several implications in the various interacting contexts. Below is an overview of the key aspects of the following five macro-categories:

1. Ability to recover
2. Type of functioning that characterizes the individual
3. Capacity to bounce back
4. Dynamic process evolving over time
5. Positive adaptation to life conditions.

### 3.1. Ability to Recover

Many authors focused on what makes people capable of dealing with the adversities of life, traumas, and stressors, trying to understand why some manage to recover after having experienced tragic events or particularly significant losses. In this regard, resilience has been defined as the ability to recover despite adverse conditions, looking ahead through a dynamic process of adaptation supported by a deeper knowledge of oneself and influenced by personal characteristics, family, and social resources (see Table 1).

### 3.2. Type of Functioning That Characterizes the Individual

Resilience is described in the literature as a peculiar response of the individual identified through the use of their personal characteristics to face difficult conditions. Since it is assumed that serious adversities destabilize most people, resilient functioning in such situations is considered extraordinary. It manifests itself in adaptive attitudes and behaviors that allow one to remain psychologically healthy—or even foresee personal growth—after exposure to stressful life events. The capacity for positive adaptation is given by specific personal attitudes and qualities promoting balance in the face of change (see Table 2).

### 3.3. Capacity to Bounce Back

Many authors define psychological resilience as the ability to recover at the same time as the development of one’s resources and potential in the face of difficulties or stressful events. Understood in this way, the resilience construct is configured as an attitude to adopt effective negotiation strategies that allow one to confront adversity and to bounce back from the negative experience by promoting a process of personal growth (see Table 3).

### 3.4. Dynamic Process Evolving Over Time

In the literature, it also emerges that coping with situations perceived as adverse or changeable occurs through a dynamic process of adaptation, influenced by personal characteristics, family, and social resources. Resilience should therefore be understood not only as a personal attribute that can lead to success, but also as a dynamic interaction associated with adaptability and a positive history of functioning after adversities (see Table 4).

### 3.5. Positive Adaptation to Life Conditions

Resilience is also referred to as the ability to deal with stress conditions. The processes of psychological resilience have to do with the cognitive evaluation carried out by the subject, which regulates the possibility of finding effective forms of adaptation. The thought processes, the emotional and behavioral responses through which resilient subjects build their personal vision of reality, give rise to decisions and behaviors that allow them to adapt to stressful or adverse conditions (see Table 5).

## 4. Discussion

Our literature review shows that psychological resilience is described in different ways. This discrepancy in terminology can hinder a shared definition of the construct, which limits comparisons among the research results and makes objective measurement difficult. Based on our analysis of the literature, we identified five macro-categories which, on the one hand, summarize the recurring elements in the definition of resilience across the works examined; on the other, they highlight the multidimensionality of the construct.

Over the years, several authors described resilience as *‘ability to recover’*. However, this expression has been understood in various ways. Specifically, some authors define resilience in terms of ability to recover from trauma, stress, or deprivation; others understand it as the ability to remain well despite the difficulties or to recover completely and quickly. The recovery is also intended as a return to a state of balance, as a posttraumatic growth ability, or as learning of useful skills for overcoming future risks. Additional authors describe resilience in terms of recovery by using protective barriers to stress. In summary, the ability of recovery represents the tendency of the individual to maintain their own internal balance despite the experience of traumatic events or stressful conditions. Moreover, the capacity for resilience indicates the ability to deal positively with traumatic events and to reorganize in the face of difficulties.

We found that resilience was also described in literature as a ‘*Type of functioning that characterizes the individual’.* The resilience functioning was intended as an ability both to maintain good levels of psychological and physical health and to return to a state of balance despite adversities. In this context, a reference to resilient qualities emerges in terms of individual features or personality traits such as robustness and resourcefulness, with some authors affirming that these qualities are innate and influenced by the external environmental only in a limited way. The functional adaptation to adverse conditions can be facilitated or hindered by the interaction between internal protective and risk factors. In this regard, literature has recently turned towards a concept of resilience understood as a complex phenomenon in which several factors come into play, including innate personality traits, personal purposes, and the external and psychosocial context. By exploring the protective factors that enable the implementation of resilient behaviors, we can identify the following categories: Cognitive flexibility, positive affect and optimism, humor, acceptance, active coping, religion or spirituality, altruism, social support, role models, exercise, capacity to recover from negative events, and stress inoculation. In summary, what determines resilience are the personal qualities identified as protective factors despite stressful or traumatic events.

A third group of definitions, albeit small, refers to resilience as ‘*capacity to bounce back’*. Although this description of resilience seems to be like that of the ability of adaptation, we believe that it deserves to be included in another category as it emphasizes a particular response mode, namely referring to some specific characteristics. Thus, resilience as *capacity to bounce back* outlines the ability to persist and grow when faced with stressors, to cope despite adversity, and to bounce from negative experience. Resilience refers to having good outcomes despite adversity and risk, and could be described in terms of preserving the same level of outcome or rebounding back to that level after an initial setback. More specifically, resilience as ‘bouncing back’ could involve either rebounding after adversity or affectively adapting to adversity.

The resilience construct over the years has also been identified as a ‘*dynamic process evolving over time*’. Specifically, dynamic process here means firstly the interaction between internal and external protective factors that act to modify the personal effects of an adverse event. Secondly, it refers to the interaction between personal characteristics, biological processes, and family and social resources that can promote or hinder resilient processes. Within this macro-category, it is possible to identify several shades of meaning of the term resilience. Some authors define it as a vital motivational force of the individual that foresees an attitude of continuous struggle and an inclination not to surrender before difficulties, or as a force that can fluctuate over time and push the person to grow through the adversities or interruptions of their life trajectory. Others emphasize the ability to promote developmental processes and identify resilience with a dynamic mental state that can adapt to changing circumstances. Resilience is also defined as a process through which individuals survive or even grow in the face of adversity. It involves both a set of qualities or internal traits, such hardiness or high self-efficacy, and external factors, such as social support, that promote coping skills.

Given the great attention paid by the literature to the meaning of resilience as a capacity for adaptation, we decided to include this definition in a specific category, namely ‘*positive adaptation to life condition’.* In describing resilience in these terms, authors emphasize different aspects of the phenomenon. Some describe it as the ability to promote positive adaptation despite exposure to adverse, stressful, or traumatic conditions. Others define it as an adaptive attitude and behavior that allows one to remain psychologically healthy, or even to thrive until a post-traumatic growth after being exposed to stressful events. Thus conceived, resilience implies that emotional resources are used to adapt to adversity; therefore, it can also be described as the process that links resources (adaptive capacities) to results (adaptation).

Other authors focus attention on the duration of the phenomenon. In particular, some describe resilience as a temporary phenomenon, subject to fluctuations based on life circumstances and the stage of development; for others it represents a stable trajectory of operation over time.

The analysis of the literature shows that the term resilience does not have a single meaning and takes on different nuances depending on the perspective from which it is analyzed. In the field of resilience studies, there is a heated debate among scholars, in particular between those who consider resilience as a trait of personality that is fixed and stable over time and therefore measurable (‘type of functioning that characterizes the individual’) and those who do not consider it a personality trait but rather as ‘dynamic process evolving over time’, which refers to the interaction between protective factors and risk factors. Specifically, the effects of protective factors such as ‘type of functioning’ are detectable only in the presence of the stressful events, and their role is to modify the response despite adversity. The ‘dynamic process’ is instead described as the interaction of a constellation of variables that allow the reduction of the impact with the risk conditions and thus effectively dealing with the adverse condition. In the ‘recovery category’, it is also possible to identify the attitude toward a gradual return to an initial state—staying well and maintaining an effective functioning—despite the destabilization caused by an adverse event that has a significant impact on the person. Therefore, it is understood as a return to an initial state of balance. The ‘capacity to bounce back’ instead emphasizes the possibility of a personal growth despite difficulties, changes, or traumatic events rather than a return to an initial state of mind. Literally, ‘bounce back’ means rebound and change of direction. It refers to the tendency to be persevering and not to give up, assuming an attitude of openness to change.

We consider the fifth category, *‘positive adaptation to life conditions’*, as transversal to the other four that we have identified because the concept of effective adaptation to life events is implicit in each of them. Nevertheless, we have established it as a different category from the others since many definitions in the literature focus on the concept of positive adaptation to define resilience. As can be seen by comparing the tables, the category that identifies resilience as *‘positive adaptation’* includes many definitions.

Summarizing all the categories, the processes of psychological resilience make it possible to face events by maintaining and enhancing one’s resources to the point of producing personal strengthening and a positive reorganization of one’s biographical history. Therefore, the use of resilient attitudes makes it possible to construct and rebuild one’s life path, to re-establish a new balance by producing change in oneself and reacting positively in the face of difficulties, transforming them into opportunities for growth.

From the study that we conducted, it was interesting to observe the multidimensionality of the resilience construct, which has been described in the literature from different points of view, in some cases with common characteristics. The revision of the definitions of resilience has allowed us to better clarify this term and to propose a broader definition characterized by the elements that we have supposed as being more indicative of the resilient attitude.

According to the results of our study, resilience should be considered as a ‘competence’ present in each individual or organization, thanks to which it is possible not to succumb to adverse events, but to react and to reach, or to return, to a state of equilibrium.

The importance of the work we carried out thus lies in having observed that resilience resources are considered as ‘skills’ that should be present and functional both on an individual level, for example in professional practice or in social relations, and in organizations.

Moreover, being a dynamic process, resilience can be implemented in order to promote a continuous growth of the person and the environment.

Getting to know more about the resilience construct also makes it feasible to structure training pathways focused on resilient human qualities as tools aimed at fostering an attitude of openness to change.

Regarding the clinical setting, the analysis of the resilience construct we carried out through the review is also a useful starting point with reference to the identification of models of psychological intervention aimed at enhancing individual resources and abilities in order to support the attitude of facing adverse situations while maintaining an adaptive functioning.

However, it is necessary to underline that this study has some limitations that we hope to fill in a future research. More specifically, a limitation refers to the methodology of selection of the articles to be analyzed. The use of the keyword ‘definition’ inserted in both of the accessed search engines has excluded some studies which could be useful to further widen the analysis of the resilience construct. Moreover, extending the study to other databases, as well as considering scientific articles written before 2002, would allow us to obtain a greater number of works and to identify probable further definitions of the term resilience. Finally, the review focused on resilience as a psychological construct, thereby excluding the possibility of accounting for nuances of the term other than the personal one that was investigated.

## 5. Conclusions

The analysis of the literature has made it possible to identify multiple definitions of psychological resilience. As proposed in our discussion of the results, the concept of resilience can be defined by focusing attention on different contents that describe it in a different way. Based on the previously discussed results, we propose our own definition of psychological resilience that takes into account the transversal elements found in the definitions we analyzed in order to proceed towards ‘a conceptual unification’ of the term.

According to the literature search that we carried out, it can be affirmed that psychological resilience is the *ability to adapt positively to life conditions*. It is a *dynamic process* evolving over time that implies a *type of adaptive functioning* that specifically allows us to face difficulties by *recovering* an initial balance or *bouncing back* as an opportunity for growth.

We believe that resilience is the ability to maintain one’s orientation towards existential purposes despite enduring adversities and stressful events. It foresees an attitude of persistence before the obstacle and openness to change. This concept can be understood as the ability to deal with the difficulties experienced in the different areas of one’s life with perseverance, maintaining a good awareness of oneself and one’s own internal and parallel coherence by activating a personal growth project. This persevering attitude makes it possible to activate one’s own resources to recover after having experienced adverse conditions, re-establishing the state of personal balance. In our definition, the term ‘purpose’ refers to long-term objectives and the overall objective regarding existence in its complexity. The latter varies from individual to individual according to their life commitments (vocational, affective, social, professional, etc.). Broadly speaking, through the acts of resilience related to partial ends, the individual becomes more and more persistent in the orientation towards their personal fulfillment. Our attempt at conceptual clarification of the term resilience, in also highlighting that specific skills and individual characteristics are necessary for a good maintaining of one’s own orientation towards existential purposes, will be a useful starting point for further research aimed at deeply exploring resilience resources and at identifying effective training strategies to support them.

## Figures and Tables

**Table 1:** Ability to recover.

Reference Articles	Definition Proposed	Citation Source
Stephens [23]	*Resilience is the capacity to recover from extremes of trauma, deprivation, threat, or stress.*	Atkinson [24]
Barber [25]	*Resistance referring to maintained functioning under stressful conditions and resilience describing quick or full recovery from significant decrements in functioning upon exposure to stress.*	Bonanno [26]; Masten [10]
Barber [25]	*Resilience as intrinsic recovery, a fundamental characteristic of normal coping, not a sign of exceptional strength.*	Bonanno [26]
Caldeira [17]	*Resilience is the ability to recover from perceived adverse or changing situations, through a dynamic process of adaptation, influenced by personal characteristics, family, and social resources, and manifested by positive coping, control, and integration.*	Caldeira [18]
Whitson [27]	*Resilience has been defined as the capacity to remain well, recover, or even thrive in the face of adversity.*	Hardy [28]
Okvat [29]	*By resilience, we mean the capacity to sustain well-being and recover fully and rapidly from adversity.*	Zautra [30]
De Terte [31]	*Psychological resilience has been defined as the ability of an individual to rebound or recover from adversity.*	Leipold [32]
De Terte [31]	*Resilience has been defined as the ability of an individual to recover quickly from the psychological effects of an adverse event.*	Bonanno [33]
Kokufu [34]	*Psychological resilience is a mental quality that leads to adaptive recovery in difficult situations despite the feeling of pain.*	Kokufu [34]
Vahia [35]	*Resilience is broadly defined in physiological terms as the ability to return to homeostasis in the presence of stressful experiences that would be expected to bring about negative effects.*	Rutter [36]
Stainton [37]	*The term resilience is used in the literature for different phenomena ranging from prevention of mental health disturbance to successful adaptation and swift recovery after experiencing life adversities and may also include post-traumatic psychological growth.*	Rutten et al. [38]
Stainton [37]	*It has also been hypothesized that resilience may result from the experience of prior stresses or adversities. Circumstances which are stressful enough to challenge, but not overwhelm, the individual can provide the opportunity to learn skills or identify attributes which can help the individual to overcome future risks.*	Harris et al. [39]
Johnston [40]	*Resilience has been referred to as a kind of plasticity that influences the ability to recover and achieve psychosocial balance after adverse experiences and as the ability to bounce back in the face of adversity. Resilience in older people has been described as the ability to achieve, retain, or regain physical or emotional health after illnesses or losses.*	Lundman [41]
Dias [42]	*Resilience is not invulnerability to stress, but, rather, the ability to recover from negative events.*	Cowan [43]
Barber [25]	*Basic conceptualizations of resilience (particularly, resistance) imply that it reflects uncommon imperviousness to expected injury or an unusual ability to quickly recover from it.*	Barber [44]
Davydov, [19]	*Some researchers describe mental resilience in terms of quick and effective recovery after stress. This parallels somatic recovery mechanisms after pathogen invasion through external and internal protective barriers, and describes the ability to ‘spring back’ to initial levels of mental, emotional, and cognitive activity after an adversity (such as functional limitation, bereavement, marital separation, or poverty).*	Tugade [45]
Dulin [46]	*Defined resilience as the “ability to resist negative psychological responses when confronted with stress or trauma.*	Pecoraro [47]

**Table 2:** Type of functioning that characterizes the individual.

Reference Articles	Definition Proposed	Citation Source
Barber [25]	*Resilience, by definition, is a unique, nonnormative type of functioning that can be exhibited only in the face of adversity. Because severe adversity is presumed to disable most people, resilient functioning in such contexts is viewed as extraordinary. As the argument goes, this would be the case especially in severely adverse contexts such as war and other forms of violent political conflict wherein simply escaping psychopathology would qualify as resilience.*	Barber [25]
Barber [25]	*Related is the debate about whether resilience should be considered as resistance or recovery. For some, rather than revealing competent adjustment, the construct describes a distinctive response in the face of challenge or risk that is variously characterized as resisting, escaping, being less vulnerable, not struggling as much as others, or having a heightened ability to handle stress.*	Hoge [48]; Westpahl [49]; Wexler [50]
Çuhadar [51]	*Psychological resilience is defined as the ability of an individual to successfully overcome negative conditions and adapt to them even when faced with difficult conditions such as serious health problems, and is a personal property as a source of resistance when faced with stressful life events.*	Luthar [15]; Reis [52]; Terzi [53]; Öz [54]; Basim [55]; Wright [56]; Schumacher [57]
McAllister [58]	*Resilience refers to one’s ability to deal with stress and adversity and is influenced by genetic, epigenetic, developmental, neurochemical, and psychosocial factors.*	Connor [13]; Evers [59]; Karoly [60]; Wu [61]
Patel [18]	*Resilience as an ability of adults to “maintain relatively stable, healthy levels of psychological and physical functioning”.*	Bonanno [62]
Johnston et al. [40]	*Resilience is the ability to maintain healthy levels of function over time despite adversity or to return to normal function after adversity.*	Bonanno [63]; Bonanno [64]; Costanzo [65]; Bonanno [33]; Scali [66]; Lam [67]; Taylor [68]
Sharpley [69]	*Psychological resilience is an intervention or buffer variable between stress and depression, possibly working by an active physiological process that reduces autonomic responses to stressors.*	Luthar Cicchetti [4]; Charney [70]
Sudom [71]	*Resilience can be viewed as a personal characteristic or set of characteristics that protects individuals from the adverse effects of stress on well-being.*	Connor [13]; Luthar, [15]
Garcia-Dia [72]	*Defined resilience as the ability of adults in otherwise normal circumstances, who were exposed to an isolated and potentially highly disruptive event, to maintain relatively stable and healthy levels of psychological and physical functioning and the capacity for generative experiences and positive emotions.*	Bonanno [62]
Ungar [73]	*Resilience is ‘‘an interactive concept that is concerned with the combination of serious risk experiences and a relatively positive psychological outcome despite those experiences’’.*	Rutter [74]
De Terte [31]	*The ability of an individual to remain psychologically healthy or stable despite the fact that they have been exposed to an adverse event.*	Bonanno [62]
Earvolino-Ramirez [75]	*The literature on ego-resiliency refers to personal characteristics of the individual as encompassing a set of traits reflecting general resourcefulness and sturdiness of character.*	Block Block [76]
Patel [18]	*Resilience “as an attribute (e.g., ability, capacity), a process, and/or an outcome associated with successful adaption to and recovery from adversity” and that it “differs depending on context and purpose”.*	Pfefferbaum [77]
Davydov [19]	*Resilience (or ‘resiliency’) as an individual trait, or an epiphenomenon of adaptive temperament.*	Ong [78]; Wachs [79]
Harvey [80]	*Resilience was largely determined by innate factors, and was therefore relatively unaffected by development or by interaction with the environment.*	Rutter [81]
Stephens [23]	*We describe a resilient individual as someone who has not only survived adversity but has also learned from the experience with resulting personal growth.*	McAllister [82]
Barber [25]	*Resilience refers explicitly and exclusively to functioning in contexts of substantial risk or adversity.*	Rutter [36]
Barber [25]	*Resilience is a unique form of competent functioning that can only be apparent in the face of considerable adversity.*	Rutter [83]
De Terte, [31]	*Resilience is the ability to maintain psychological and physical health despite exposure to a traumatic event.*	Bonanno [62]
Brodsky [84]	*Resilience consists of internal, local level goals that are aimed at intrapersonal actions and outcomes—adapting, withstanding, or resisting the situation as it is. Empowerment is enacted socially—aimed at external change to relationships, situations, power dynamics, or contexts—and involves a change in power, along with an internal psychological shift.*	Cattaneo [85]
Kim-Cohen [86]	*Resilience is theorized to result from a dynamic interplay among multiple factors that threaten adaptive functioning, as well as multilevel factors that protect against adversity and promote positive adjustment.*	Cicchetti [87]; Luthar [88]; Masten [89]; Rutter [74]
Cuhadar [51]	*Psychological resilience depends on various factors involving cognitive flexibility, positive affect and optimism, humor, acceptance, active coping and religion/ spirituality, altruism, social support, role models, exercise, capacity to recover from negative events, and stress inoculation.*	Southwick [90]
Hilliard [91]	*Resilience is the demonstration of emotional, behavioral, or health outcomes that match or surpass normative developmental milestones, behavioral functioning, or emotional well-being, despite exposure to the substantial challenges of living with and managing a medical or developmental condition. These resilient outcomes should first focus on explicitly positive experiences or the maintenance of a typical trajectory, but could also include the absence of negative experiences, such as low levels of distress or dysfunction.*	Hilliard [92]
Graber [93]	*Resilience is associated with lowered psychological distress and health-promoting lifestyles.*	Black [93]; Campbell-Sills [94]
Tan [95]	*Resilience can potentially refer to pre-existing personality traits, the dynamic process of adaptation, a psychosocial outcome, or a mixture of all three.*	Luthar [15]; Bonanno [96]; Southwick [97]
Tan [95]	*Specific qualities comprising resilience have been identified including optimism, active coping skills and maintaining a social network.*	Iacoviell [98]
Eshel [99]	*Resilience has thus been defined as a stable trajectory of healthy functioning after a highly adverse event.*	Southwick [97]
Eshel [99]	*Resilience has thus been defined as the balance of individual strength (protective factors) and vulnerability (risk factors) following an adversity or a traumatic event.*	Eshel [100]; Eshel [101]
Eshel [99]	*Resilience represents an integration of strength and vulnerability, and that understanding adaptation to adversities requires a concurrent examination of protective processes and risk factors.*	Masten [102]
Stainton [37]	*Healthy, adaptive, or integrated positive functioning over the passage of time in the aftermath of adversity.*	Southwick [97]
Stainton [37]	*In the context of exposure to significant adversity, resilience is both the capacity of individuals to navigate their way to the psychological, social, cultural, and physical resources that sustain their wellbeing, and their capacity individually and collectively to negotiate for these resources to be provided and experienced in culturally meaningful ways.*	Ungar [103]
Stainton [37]	*Resilience is a dynamic capability which can allow people to thrive on challenges given appropriate social and personal contexts.*	Howe [104]
Dulin [46]	*Resilience as a mechanism for the protective effects of conscientiousness on health outcomes.*	O’Cleirigh [105]
Dulin [46]	*Defined resilience as “a combination of personality characteristics and successful coping that allows an individual to function adaptively in the face of or following adversity.”*	Dale [106]
Casale [107]	*Resilience is broadly defined as a protective factor that makes people less vulnerable to future adverse life events.*	Ayed [108]
Li [109]	*Resilient people have the ability to adjust and cope successfully in the face of adversity, exhibiting a stable trajectory of healthy functioning across time and the capacity for positive emotions after having experienced stressful life events.*	Bonanno [110]
Sharpley [69]	*Psychological resilience defined as a set of specific behavioral or attitudinal skills which help an individual cope effectively with stress and avoid becoming depressed.*	Von Ammon [111]; Bitsika [112]; Sharpley [113]
De Terte, [114]	*Psychological resilience is as a combination of cognitions, behaviors, and environmental factors. These factors are optimism, adaptive coping, adaptive health practices, and social support from colleagues.*	De Terte, [31]

**Table 3:** Capacity to bounce back.

Reference Articles	Definition Proposed	Citation Source
Silverman [115]	*Resilience is defined in the positive psychology literature as the human capacity to persist, bounce back, and flourish when faced with stressors.*	Bonanno [62]
Chen [116]	*Resilience is the capacity to adapt to and bounce back from adversity and stressful events.*	Davidson et al. [117]; Prince-Embury [118]
Brush [119]	*Resilience as the ability to bounce back or cope successfully despite substantial adversity.*	Earvolino-Ramirez [75]
Netuveli [120]	*Resilience is having good outcomes despite adversity and risk and could be described in terms of preserving the same level of the outcome or rebounding back to that level after an initial setback. Using the latter definition, resilience as “bouncing back”. Resilience could involve either rebounding after adversity.*	Garmezy, [20]
Violanti [121]	*The term resilience is often used to imply an ability to bounce back. Consequently, the definition adopted here embodies the notion of adaptive capacity.*	Klein [122]
Earvolino-Ramirez [76]	*Resilience, the ability to bounce back or cope successfully despite substantial adversity.*	Rutter [8]
Kalisch [123]	*The process of effectively negotiating, adapting to, or managing significant sources of stress or trauma. Assets and resources within the individual, their life, and environment facilitate this capacity for adaptation and “bouncing back” in the face of adversity. Across the life course, the experience of resilience will vary.*	Windle [124]
Xing [125]	*Psychological resilience is defined as an individual’s ability to effectively adapt to and rebound from negative experience.*	Lazarus [126]
Sharpley [69]	*Various aspects of this construct of psychological resilience have been identified, including the ability to rebound from disappointments, positive adjustment behaviors in adverse circumstances, or simply successful adaptation to challenging life stressors.*	Brooks [127]; Tedeschi [128]; Alvord [129]

**Table 4:** Dynamic process evolving over time.

Reference Articles	Definition Proposed	Citation Source
Dias [42]	*Resilience may be defined as a dynamic process involving the interaction between both risk and protective factors, internal and external to the individual, that act to modify the effects of an adverse life event.*	Brandão [130]; Yunes [131]
Dias [42]	*Resilience should be understood not only as a personal attribute that may lead to success, but also as the dynamic interaction between biological and psychosocial processes.*	Dias [42]
Caldeira [17]	*We propose a definition of resilience which is the ability to recover from perceived adverse or changing situations, through a dynamic process of adaptation, influenced by personal characteristics, family, and social resources, and manifested by positive coping, control, and integration.*	Caldeira [17]
Kim-Cohen [86]	*Resilience is theorized to result from a dynamic interplay among multiple factors that threaten adaptive functioning, as well as multilevel factors that protect against adversity and promote positive adjustment.*	Cicchetti [87]; Luthar [88]; Masten [89]; Rutter [36]
Stephens [23]	*Resilience is “an ongoing process of struggling with hardship and not giving up”.*	Gillespie [132]
Garcia-Dia [72]	*Resilience can occur either as a process or as a motivational life force that can be developed in individuals.*	Haase [133]
Davydov [19]	*Resilience as a process or force that drives a person to grow through adversity and disruption.*	Jacelon [134]; Richardson, [135]; Richardson [7]
Patel [18]	*Resilience is “a process or the attainment of positive outcomes at the individual, family, and community levels despite adversity (e.g., natural disaster, terrorist attack).*	Lemyre [136]
Kim-Cohen [86]	*Resilience is conceptualized as dynamic with the understanding that adjustment can fluctuate over time in response to a stressor.*	Masten [102]; Masten Narayan [137]
Davydov [19]	*Emotional resilience has been used as the process linking resources (adaptive capacities) to outcomes (adaptation).*	Norris [138]
Levine [139]	*Patterson’s perspective that family resilience is an “ongoing, emergent process”. Resilience was manifest in the process of moving from the position of “knowing” through listening to others to “knowing” developed in the context of listening to self.*	Patterson [140]
Kim-Cohen [86]	*Resilience is dynamic and interactive in that it is a process stimulated by the presence of adversity rather than simply the balance of risk versus protective factors.*	Rutter [141]
Brush [119]	*Resilience is the process as “the capability to adapt better than expected in the face of significant adversity or risk”.*	Tusaie [142]
Brush [119]	*Resilience implies a process of hurdling resistance and, in doing so, gaining strength against future stressors, challenges, crises, or trauma, much like a microbe develops resilience over time to an antibiotic and ultimately adapts to and survives its environmental conditions. Adaptation and survival are thus consequences of resiliency while resiliency is an important individual characteristic in the process of overcoming.*	Hernandez [143]
Lee [144]	*Resilience as an active process that develops internal resources for coping with stress.*	Woodgate [145]
Cuhadar [51]	*Resilience is a dynamic process related to an individual’s capacity to cope with difficult or stressful experiences and the ability to psychologically overcome adversity.*	Luthar [15]; Masten [146]; Basim [55]; Wright [56]; Sharpley [69]
Karoly [60]	*Resilience is considered both functional and dynamic, in that it implies the effective performance of life tasks by virtue of a complex interaction between varied risk and protective factors.*	Luthar [14]; Olson [147]
Takahashi [148]	*Resilience is defined as “a dynamic process encompassing positive adaptation within the context of significant adversity.*	Luthar [15]
Whitson [27]	*Resilience is a process associated with adaptive capacities and a positive history of functioning and adaptation after adversities.*	De Alfieri [149]
Paletti [150]	*Resilience as ‘‘a set of qualities that foster a process of successful adaptation and transformation’’ in the face of adversity. Resilience may be seen as prerequisite to recovery; by engaging in resilient behaviors, bereaved individuals may work toward the self-transformation inherent in successful adaptation to loss.*	Benard [151]
Earvolino-Ramirez [75]	*Resilience is a dynamic developmental process.*	Luthar [152]
Pangallo [153]	*Resilience is best defined as a process characterized by a complex interaction of internal and external resources moderated by developmental influences.*	Masten [154]; Rutter [155]; Werner [156]; Windle [124]
Ungar [73]	*I defined resilience as the capacity of both individuals and their environments to interact in ways that optimize developmental processes. Specifically, research shows that in situations of adversity, resilience is observed when individuals engage in behaviors that help them to navigate their way to the resources they need to flourish.*	Ungar [157]
Dias [42]	*Resilience is a process associated with adaptive capacities and a positive history of functioning and adaptation after adversities. This dynamic process involves the interaction between biological and psychosocial factors, which makes its investigation more complex.*	Dias [42]
Tan [95]	*Resilience refers to a dynamic process of positive adaptation within the context of adversity.*	Luthar [15]
Eshel [99]	*Resilience is a dynamic state of mind that may change due to changing circumstances which will modify the existing balance of individual protective factors and risk factors.*	Ungar [103]
Stainton [37]	*“Resilience is the process of effectively negotiating, adapting to, or managing significant sources of stress or trauma.”*	Windle [124]
Graber [92]	*Psychological resilience is a psychosocial developmental process through which people exposed to sustained adversity experience positive psychological adaptation.*	Luthar [14]; Rutter, [74]
Dulin [46]	*Resilience resources are also viewed here as processes that buffer against and are potentially more malleable to intervention than some of the aforementioned adversities at the individual, interpersonal, and neighborhood levels.*	Dale [158]; De Santis [159]; Kent [160]; Steinhardt [161]

**Table 5:** Positive adaptation to life conditions.

Reference Articles	Definition Proposed	Citation Source
Dias [42]	*Resilience was defined as positive adjustment in the case of adversity.*	Bekhet [162]; Fernández-Lansac [163]; O’Rourke [164]; Bull [165]; Fitzpatrick [166]; Garces [167]; Wilks [168]
Hilliard [91]	*Resilience: achieving one or more positive outcomes despite exposure to significant risk or adversity.*	Hilliard [169]
Davydov [19]	*Resilience can be seen as synonymous with reduced ‘vulnerability’ with ability to adapt to adversity.*	Hofer [170]; Schneiderman, [171]
Brodsky [84]	*Resilience* is successful adaptation despite risk and adversity.	Masten, [10]
Lee [172]	*Defining resilience as the process of, capacity for, or outcome of successful adaptation despite challenging or threatening circumstances.*	Masten [173]
Thomas [174]	*Resilience is defined as the ability to overcome adversity and includes how one learns to grow stronger from the experience.*	McAllister [175]
Thompson [176]	*Psychological resilience is characterized by the ability to successfully adapt to stressful events in the face of adverse conditions.*	Norman [177]
Pangallo [153]	*Resilience is a temporal phenomenon, and as such, positive adaptation is likely to fluctuate according to circumstances and life stage.*	Pangallo [153]
Garcia-Dia [72]	*Resilience as adaptation and adjustment that occurs despite multiple personal and social losses.*	Rabkin [178]
Brush [119]	*Resilience is the capability to adapt better than expected in the face of significant adversity or risk.*	Tusaie [142]
Davydov [19]	*Resilience can be viewed as an epiphenomenon of adaptive temperament.*	Wachs [79]
Li [108]	*Resilient people have the ability to adjust and cope successfully in the face of adversity, exhibiting a stable trajectory of healthy functioning across time and the capacity for positive emotions after having experienced stressful life events.*	Bonanno [110]
Chen [116]	*Resilience is the capacity to adapt to and bounce back from adversity and stressful events.*	Davidson [117]; Prince-Embury [118]
Caldeira [17]	*Resilience is considered both as a psychological and physical aspect of coping with stress.*	Hart [179]
Garcia-Dia [72]	*Resilience was in fact quite common rather than uncommon as had been proposed by earlier researchers, and a fundamental feature of normal coping skills as manifested by seeking social support from others, moving forward with life and accepting your circumstances with hope.*	Masten, [10]
Eisenach [180]	*Resilience is a measure of coping ability, hardiness, and the ability to thrive in the face of adversity.*	Vaishnavi [181]
Lee [144]	*Resilience as an active process that develops internal resources for coping with stress.*	Woodgate [145]
Lee [172]	*Resilience as a capacity refers to an individual’s capacity for adapting to changes and stressful events in a healthy way.*	Catalano [182]
Chen [116]	*Resilience is essentially a capacity of positive adaptation after exposure to social and psychological adversity.*	Prince-Embury [118]
Whitson [27]	*Resilience as a psychological construct, referring to adaptive attitudes and behaviors that allow one to remain psychologically sound, or even thrive, after being exposed to stressful life events.*	Luthar [15]; Wagnild [183]
Li [109]	*Resilience can be used to represent an individual’s successful adaptation to trauma.*	Wang [184]
Miller [185]	*Resilience as a personality characteristic that minimizes the negative effects of stress and promotes adaptation.*	Wagnild [186]
Patel [18]	*Resilience is not a process, it is not a management system standard, nor is it a consulting product. Resilience is a demonstrable outcome of an organization’s capability to cope with uncertainty and change in an often volatile environment. Resilience is thus a product of an organization’s capabilities for interacting with its environment.*	Gibson [187]
Horn [188]	*Resilience is broadly defined as the dynamic ability to adapt successfully in the face of adversity, trauma, or significant threat. Resilience is complex and might be best conceptualized on a continuum, with the potential for it to change across an individual’s lifespan.*	Southwick [97]
Cosco [189]	*Resilience involves positively adapting to adverse events.*	Luthar [190]; Rutter [155]
Harvey [80]	*Resilience as ‘‘manifested competence in the context of significant challenges to adaptation or development’’.*	Masten [191]
Davydov [19]	*Emotional resilience has been used as a concept to imply the flexible use of emotional resources for adapting to adversity or as the process linking resources (adaptive capacities) to outcomes (adaptation).*	Waugh [192]; Norris [138]
Johnston [40]	*Resilience can be conceptualized as the process of achieving unexpected positive outcomes in adverse conditions, as opposed to an individual trait.*	Taylor [68]
Dias [42]	*Resilience is a process related to adaptive capacities or to a positive trajectory of functioning and adaptation after a traumatic situation.*	Fitzpatrick [166]; Garces [167]; Norris [138]; Wilks [168]
Eshel [99]	*Resilience has thus been defined as “the potential of manifested capacity of a dynamic system to adapt successfully to disturbances that threaten the function, survival, or development of the system”.*	Masten [192]
Stainton [37]	*Resilience appears to be a common phenomenon that results in most cases from the operation of basic human adaptational systems. If those systems are protected and in good working order, development is robust even in the face of severe adversity.*	Masten [10]
Dulin [46]	*Resilience resources as positive psychological, behavioral, and/or social adaptation in the face of stressors and adversities that draws upon “an individual’s capacity, combined with families’ and communities’ resources to overcome serious threats to development and health”.*	Fletcher [1]; Earnshaw [193]; Unger [194]

## References

[1] Fletcher D., Sarkar M. (2013). Psychological resilience: A review and critique of definitions, concepts, and theory. Eur. Psychol..

[2] Grinker R.R., Spiegal J.P. (1963). Under Stress.

[3] Jackson D., Firtko A., Edenborough M. (2007). Personal resilience as a strategy for surviving and thriving in the face of workplace adversity: A literature review. J. Adv. Nurs..

[4] Luthar S.S., Cicchetti D. (2000). The construct of resilience: Implications for interventions and social policies. Dev. Psychopathol..

[5] Vanistendael S., Lecomte J. (2000). Le Bonheur est Toujours Possible: Construire La Resilience.

[6] Richardson G.E., Neiger B.L., Jensen S., Kumpfer K.L. (1990). The resiliency model. Health Educ..

[7] Richardson G.E., Waite P.J. (2002). Mental health promotion through resilience and resiliency education. Int. J. Emerg. Ment. Health.

[8] Rutter M. (1993). Resilience: Some conceptual considerations. J. Adolesc. Health.

[9] Werner E.E., Smith R.S. (1992). Overcoming the Odds: High Risk Children from Birth to Adulthood.

[10] Masten A.S. (2001). Ordinary magic: Resilience processes in development. Am. Psychol..

[11] Antonovsky A. (1987). The salutogenic perspective: Toward a new view of health and illness. Advances.

[12] Miller T.W. (1988). Advances in understanding the impact of stressful life events on health. Psychiatr. Serv..

[13] Connor K.M., Davidson J.R. (2003). Development of a new resilience scale: The Connor-Davidson resilience scale (CD-RISC). Depress. Anxiety.

[14] Flach F. (1988). Resilience: Discovering a New Strength at Times of Stress.

[15] Luthar S.S., Cicchetti D., Becker B. (2000). The construct of resilience: A critical evaluation and guidelines for future work. Child. Dev..

[16] Block J., Block J.H. (2014). The role of ego-control and ego-resiliency in the organization of behavior. Development of Cognition, Affect, and Social Relations.

[17] Caldeira S., Timmins F. (2016). Resilience: Synthesis of concept analyses and contribution to nursing classifications. Int. Nurs. Rev..

[18] Patel S.S., Rogers M.B., Amlôt R., Rubin G.J. (2017). What do we mean by ‘community resilience’? A systematic literature review of how it is defined in the literature. PLoS Curr..

[19] Davydov D.M., Stewart R., Ritchie K., Chaudieu I. (2010). Resilience and mental health. Clin. Psychol. Rev..

[20] Luthar S.S. (2015). Resilience in development: A synthesis of research across five decades. Developmental Psychopathology: Volume Three: Risk, Disorder, and Adaptation.

[21] Garmezy N. (1993). Children in poverty: Resilience despite risk. Psychiatry.

[22] Rutter M.E. (1988). Studies of Psychosocial Risk: The Power of Longitudinal Data.

[23] Stephens T.M. (2013). Nursing student resilience: A concept clarification. Nurs. Forum.

[24] Atkinson P.A., Martin C.R., Rankin J. (2009). Resilience revisited. J. Psychiatr. Ment. Health Nurs..

[25] Barber B.K. (2013). Annual research review: The experience of youth with political conflict–challenging notions of resilience and encouraging research refinement. J. Child. Psychol. Psychiatry.

[26] Bonanno G.A. (2008). The human capacity to thrive in the face of potential trauma. Pediatrics.

[27] Whitson H.E., Duan-Porter W., Schmader K.E., Morey M.C., Cohen H.J., Colón-Emeric C.S. (2015). Physical resilience in older adults: Systematic review and development of an emerging construct. J. Gerontol. Ser. A Biomed. Sci. Med. Sci..

[28] Hardy S.E., Concato J., Gill T.M. (2004). Resilience of community-dwelling older persons. J. Gerontol. Med. Sci..

[29] Okvat H.A., Zautra A.J. (2011). Community gardening: A parsimonious path to individual, community, and environmental resilience. Am. J. Community Psychol..

[30] Zautra A.J., Hall J.S., Murray K.E. (2008). Resilience: A new integrative approach to health and mental health research. Health Psychol. Rev..

[31] De Terte I., Stephens C., Huddleston L. (2014). The development of a three part model of psychological resilience. Stress Health.

[32] Leipold B., Greve W. (2009). Resilience. Eur. Psychol..

[33] Bonanno G.A., Brewin C.R., Kaniasty K., La Greca A.M. (2010). Weighing the costs of disaster: Consequences, risks, and resilience in individuals, families, and communities. Psychol. Sci. Public Interest.

[34] Kokufu H. (2012). Conflict accompanying the choice of initial treatment in breast cancer patients. Jpn. J. Nurs. Sci..

[35] Vahia I.V., Chattillion E., Kavirajan H., Depp C.A. (2011). Psychological protective factors across the lifespan: Implications for psychiatry. Psychiatr. Clin..

[36] Rutter M. (2006). Genes and Behavior: Nature-Nurture Interplay Explained.

[37] Stainton A., Chisholm K., Kaiser N., Rosen M., Upthegrove R., Ruhrmann S., Wood S.J. (2018). Resilience as a multimodal dynamic process. Early Interv. Psychiatry.

[38] Rutten B.P., Hammels C., Geschwind N., Menne-Lothmann C., Pishva E., Schruers K., Van Den Hove D., Kenis G., Van Os G., Wichers M. (2013). Resilience in mental health: Linking psychological and neurobiological perspectives. Acta Psychiatr. Scand..

[39] Harris M.A., Brett C.E., Starr J.M., Deary I.J., McIntosh A.M. (2016). Early-life predictors of resilience and related outcomes up to 66 years later in the 6-day sample of the 1947 Scottish mental survey. Soc. Psychiatry Psychiatr. Epidemiol..

[40] Johnston M.C., Porteous T., Crilly M.A., Burton C.D., Elliott A., Iversen L., Black C. (2015). Physical disease and resilient outcomes: A systematic review of resilience definitions and study methods. Psychosomatics.

[41] Lundman B., Aléx L., Jonsén E., Lövheim H., Nygren B., Fischer R.S., Strandberg G., Norberg A. (2012). Inner strength in relation to functional status, disease, living arrangements, and social relationships among people aged 85 years and older. Geriatr. Nurs..

[42] Dias R., Santos R.L., Sousa M.F.B.D., Nogueira M.M.L., Torres B., Belfort T., Dourado M.C.N. (2015). Resilience of caregivers of people with dementia: A systematic review of biological and psychosocial determinants. Trends Psychiatry Psychother..

[43] Cowan P.A., Cowan C.P., Schulz M.S. (1996). Thinking about risk and resilience in families. Stress, Coping, and Resiliency in Children and Families.

[44] Barber B.K., Doty S.B. (2013). How can a majority be resilient? Critiquing the utility of the construct of resilience through a focus on youth in contexts of political conflict. Handbook of Resilience in Children of War.

[45] Tugade M.M., Fredrickson B.L. (2004). Resilient individuals use positive emotions to bounce back from negative emotional experiences. J. Personal. Soc. Psychol..

[46] Dulin A.J., Dale S.K., Earnshaw V.A., Fava J.L., Mugavero M.J., Napravnik S., Howe C.J. (2018). Resilience and HIV: A review of the definition and study of resilience. AIDS Care.

[47] Pecoraro A., Pacciolla A., O’Cleirigh C., Mimiaga M., Kwiatek P., Blokhina E., Verbitskaya E., Krupitsky E., Woody G.E. (2016). Proactive coping and spirituality among patients who left or remained in antiretroviral treatment in St Petersburg, Russian Federation. AIDS Care.

[48] Hoge E.A., Austin E.D., Pollack M.H. (2007). Resilience: Research evidence and conceptual considerations for posttraumatic stress disorder. Depress. Anxiety.

[49] Westphal M., Bonanno G.A. (2007). Posttraumatic growth and resilience to trauma: Different sides of the same coin or different coins?. Appl. Psychol..

[50] Wexler L.M., Di Fluvio G., Burke T.K. (2009). Resilience and marginalized youth: Making a case for personal and collective meaning-making as part of resilience research in public health. Soc. Sci. Med..

[51] Cuhadar D., Tanriverdi D., Pehlivan M., Kurnaz G., Alkan S. (2016). Determination of the psychiatric symptoms and psychological resilience levels of hematopoietic stem cell transplant patients and their relatives. Eur. J. Cancer Care.

[52] Reis S.M., Colbert R.D., Hébert T.P. (2004). Understanding resilience in diverse, talented students in an urban high school. Roeper Rev..

[53] Terzi S. (2008). Üniversite ög˘rencilerinin psikolojik dayanikliliklari ile algiladiklari sosyal destek arasindaki ilis¸ki. Türk Psikolojik Danis¸ma ve Rehberlik Dergisi.

[54] Öz P.D.F., Yilmaz U.H.E.B. (2009). Ruh sağlığının korunmasında önemli bir kavram: Psikolojik sağlamlık. Hacettepe Üniversitesi Hemşirelik Fakültesi Dergisi.

[55] Basim N., Çetin F. (2011). Yetis¸kinleriçin psikolojik dayaniklilik ölçeg˘inin güvenirlik ve geçerlilik çalis¸masi. Türk Psikiyatri Dergisi.

[56] Wright M.O.D., Masten A.S., Narayan A.J. (2013). Resilience Processes in Development: Four Waves of Research on Positive Adaptation in the Context of Adversity. Handbook of Resilience in Children.

[57] Schumacher A., Sauerland C., Silling G., Berdel W.E., Stelljes M. (2014). Resilience in patients after allogeneic stem cell transplantation. Support. Care Cancer.

[58] McAllister S.J., Vincent A., Hassett A.L., Whipple M.O., Oh T.H., Benzo R.P., Toussaint L.L. (2015). Psychological resilience, affective mechanisms and symptom burden in a tertiary-care sample of patients with fibromyalgia. Stress Health.

[59] Evers A.W., Zautra A., Thieme K. (2011). Stress and resilience in rheumatic diseases: A review and glimpse into the future. Nat. Rev. Rheumatol..

[60] Karoly P.L.S. (2006). Psychological “resilience” and its correlates in chronic pain: Findings from a national community sample. Pain.

[61] Wu G., Feder A., Cohen H., Kim J.J., Calderon S., Charney D.S., Mathe A.A. (2013). Understanding resilience. Front. Behav. Neurosci..

[62] Bonanno G.A. (2004). Loss, trauma, and human resilience: Have we underestimated the human capacity to thrive after extremely avversive events?. Am. Psychol..

[63] Bonanno G.A., Galea S., Bucciarelli A., Vlahov D. (2007). What predicts psychological resilience after disaster? The role of demographics, resources, and life stress. J. Consult. Clin. Psychol..

[64] Bonanno G.A., Ho S.M., Chan J.C., Kwong R.S., Cheung C.K., Wong C.P., Wong V.C. (2008). Psychological resilience and dysfunction among hospitalized survivors of the SARS epidemic in Hong Kong: A latent class approach. Health Psychol..

[65] Costanzo E.S., Ryff C.D., Singer B.H. (2009). Psychosocial adjustment among cancer survivors: Findings from a national survey of health and well-being. Health Psychol..

[66] Scali J., Gandubert C., Ritchie K., Soulier M., Ancelin M.L., Chaudieu I. (2012). Measuring resilience in adult women using the 10-items Connor-Davidson Resilience Scale (CD-RISC). Role of trauma exposure and anxiety disorders. PLoS ONE.

[67] Lam W.W., Bonanno G.A., Mancini A.D., Ho S., Chan M., Hung W.K., Or A., Fielding R. (2010). Trajectories of psychological distress among Chinese women diagnosed with breast cancer. Psychooncology.

[68] Taylor J., Jacoby A., Baker G.A., Marson A.G., Ring A. (2011). White head M: Factors predictive of resilience and vulnerability in new on set epilepsy. Epilepsia.

[69] Sharpley C.F., Bitsika V., Wootten A.C., Christie D.R. (2014). Does resilience ‘buffer’against depression in prostate cancer patients? A multi-site replication study. Eur. J. Cancer Care.

[70] Charney D.S. (2004). Psychobiological mechanisms of resilience and vulnerability: Implications for successful adaptation to extreme stress. Am. J. Psychiatry.

[71] Sudom K.A., Lee J.E., Zamorski M.A. (2014). A longitudinal pilot study of resilience in Canadian military personnel. Stress Health.

[72] Garcia-Dia M.J., DiNapoli J.M., Garcia-Ona L., Jakubowski R., O’flaherty D. (2013). Concept analysis: Resilience. Arch. Psychiatr. Nurs..

[73] Ungar M. (2013). Resilience, trauma, context, and culture. Trauma Violence Abuse.

[74] Rutter M. (2006). Implications of resilience concepts for scientific understanding. Ann. N. Y. Acad. Sci..

[75] Earvolino-Ramirez M. (2007). Resilience: A concept analysis. Nursing Forum.

[76] Block J.H., Block J., Collins W.A., Hillsdale N.J. (1980). The role of ego-control and ego-resiliency in the organization of behavior. Development of Cognition, Affect and Social Relations: The Minnesota Symposia on Child Psychology.

[77] Pfefferbaum B., Pfefferbaum R.L., Van Horn R.L. (2015). Community resilience interventions: Participatory, assessment-based, action-oriented processes. Am. Behav. Sci..

[78] Ong A.D., Bergeman C.S., Bisconti T.L., Wallace K.A. (2006). Psychological resilience, positive emotions, and successful adaptation to stress in later life. J. Personal. Soc. Psychol..

[79] Wachs T.D. (2006). Contributions of temperament to buffering and sensitization processes in children’s development. Ann. N. Y. Acad. Sci..

[80] Harvey J., DelFabbro P.H. (2004). Psychological resilience in disadvantaged youth: A critical overview. Aust. Psychol..

[81] Rutter M. Resilience: Some conceptual considerations initiatives. Proceedings of the Conference on Fostering Resilience.

[82] McAllister M Lowe J.B. (2011). The Resilient Nurse: Empowering Your Practice.

[83] Rutter M. (2012). Resilience as a dynamic concept. Dev. Psychopathol..

[84] Brodsky A.E., Cattaneo L.B. (2013). A transconceptual model of empowerment and resilience: Divergence, convergence and interactions in kindred community concepts. Am. J. Community Psychol..

[85] Cattaneo L.B., Chapman A.R. (2010). The process of empowerment: A model for use in research and practice. Am. Psychol..

[86] Kim-Cohen J., Turkewitz R. (2012). Resilience and measured gene–environment interactions. Dev. Psychopathol..

[87] Cicchetti D., Blender J.A. (2006). A multiple-levels-of-analysis perspective on resilience: Implications for the developing brain, neural plasticity, and preventive interventions. Ann. N. Y. Acad. Sci..

[88] Luthar S.S., Brown P.J. (2007). Maximizing resilience through diverse levels of inquiry: Prevailing paradigms, possibilities, and priorities for the future. Dev. Psychopathol..

[89] Masten A.S. (2012). Resilience in children. Developmental Psychology: Revisiting the Classic Studies.

[90] Southwick S.M., Vythilingam M., Charney D.S. (2005). The psychobiology of depression and resilience to stress: Implications for prevention and treatment. Annu. Rev. Clin. Psychol..

[91] Hilliard M.E., McQuaid E.L., Nabors L., Hood K.K. (2015). Resilience in youth and families living with pediatric health and developmental conditions: Introduction to the special issue on resilience. J. Pediatr. Psychol..

[92] Graber R., Turner R., Madill A. (2016). Best friends and better coping: Facilitating psychological resilience through boys’ and girls’ closest friendships. Br. J. Psychol..

[93] Black C., Ford-Gilboe M. (2004). Adolescent mothers: Resilience, family health work and health promoting practices. J. Adv. Nurs..

[94] Campbell-Sills L., Cohan S.L., Stein M.B. (2006). Relationship of resilience to personality, coping, and psychiatric symptoms in young adults. Behav. Res. Ther..

[95] Tan W.S., Beatty L., Kemp E., Koczwara B. (2019). What contributes to resilience in cancer patients? A principal component analysis of the Connor—Davidson Resilience Scale. Asia Pac. J. Clin. Oncol..

[96] Bonanno G.A., Westphal M., Mancini A.D. (2011). Resilience to loss and potential trauma. Annu. Rev. Clin. Psychol..

[97] Southwick S.M., Bonanno G.A., Masten A.S., Panter-Brick C., Yehuda R. (2014). Resilience definitions, theory, and challenges: Interdisciplinary perspectives. Eur. J. Psychotraumatol..

[98] Iacoviell B.M., Charney D.S. (2014). Psychosocial facets of resilience: Implications for preventing posttrauma psychopathology, treating trauma survivors, and enhancing community resilience. Eur. J. Psychotraumatol..

[99] Eshel Y., Kimhi S., Lahad M., Leykin D., Goroshit M. (2018). Risk Factors as Major Determinants of Resilience: A Replication Study. Community Ment. Health J..

[100] Eshel Y., Kimhi S. (2016). Determinants of individual resilience following missile attacks: A new perspective. Personal. Individ. Differ..

[101] Eshel Y., Kimhi S., Lahad M., Leykin D. (2016). Individual, community, and national resiliencies and age: Are older people less resilient than younger individuals?. Am. J. Geriatr. Psychiatry.

[102] Masten A.S. (2011). Resilience in children threatened by extreme adversity: Frameworks for research, practice, and translational synergy. Dev. Psychopathol..

[103] Ungar M. (2011). Community resilience for youth and families: Facilitative physical and social capital in contexts of adversity. Child. Youth Soc. Serv. Rev..

[104] Howe A., Smajdor A., Stöckl A. (2012). Towards an understanding of resilience and its relevance to medical training. Med. Educ..

[105] O’cleirigh C., Ironson G., Weiss A., Costa P.T. (2007). Conscientiousness predicts disease progression (CD4 number and viral load) in people living with HIV. Health Psychol..

[106] Dale S., Cohen M., Weber K., Cruise R., Kelso G., Brody L. (2014). Abuse and resilience in relation to HAART medication adherence and HIV viral load among women with HIV in the United States. AIDS Patient Care Stds..

[107] Casale R., Sarzi-Puttini P., Botto R., Alciati A., Batticciotto A., Marotto D., Torta R. (2019). Fibromyalgia Concept Resilience. Clin. Exp. Rheumatol..

[108] Ayed N., Toner S., Priebe S. (2018). Conceptualizing resilience in adult mental health literature: A systematic review and narrative synthesis. Psychol. Psychother. Theory Res. Pract..

[109] Li Y., Cao F., Cao D., Liu J. (2015). Nursing students’ post-traumatic growth, emotional intelligence and psychological resilience. J. Psychiatr. Ment. Health Nurs..

[110] Bonanno G.A., Papa A., O’Neill K. (2001). Loss and human resilience. Appl. Prev. Psychol..

[111] Von Ammon Cavanagh S., Furlanetto L., Creech S., Powell L. (2001). Medical illness, past depression, and present depression: A predictive triad for in hospital mortality. Am. J. Psychiatry.

[112] Bitsika V., Sharpley C., Aroutzidis A., Smith D. (2011). The impact of students ‘internally-’ versus ‘externally-oriented’ coping strategies upon anxiety and depression: Implications for counselling processes. Asia-Pac. J. Couns. Psychother..

[113] Sharpley C., Bitsika V., Christie D. (2012). How prostate cancer patients cope: Evaluation and refinement of the Prostate Cancer Patients’ Coping Strategies Scale. J. Men Health.

[114] De Terte I., Stephens C. (2014). Psychological resilience of workers in high-risk occupations. Stress Health.

[115] Silverman A.M., Verrall A.M., Alschuler K.N., Smith A.E., Ehde D.M. (2017). Bouncing back again, and again: A qualitative study of resilience in people with multiple sclerosis. Disabil. Rehabil..

[116] Chen X., Wang Y., Yan Y. (2016). The Essential Resilience Scale: Instrument Development and Prediction of Perceived Health and Behaviour. Stress Health.

[117] Davidson J.R.T., Payne V.M., Connor K.M., Foa E.B., Rothbaum B.O., Hertzberg M.A., Weisler R.H. (2005). Trauma, resilience and saliostasis: Effects of treatment in post-traumatic stress disorder. Int. Clin. Psychopharmacol..

[118] Prince-Embury S., Saklofske D.H. (2013). Resilience in Children, Adolescents, and Adults: Translating Research into Practice.

[119] Brush B.L., Kirk K., Gultekin L., Baiardi J.M. (2011). Overcoming: A concept analysis. Nursing Forum.

[120] Netuveli G., Wiggins R.D., Montgomery S.M., Hildon Z., Blane D. (2008). Mental health and resilience at older ages: Bouncing back after adversity in the British Household Panel Survey. J. Epidemiol. Community Health.

[121] Violanti J.M., Paton D., Johnston P., Burke K.J., Clarke J., Keenan D. (2008). Stress shield: A model of police resiliency. Emerg. Mental Health.

[122] Klein R., Nicholls R.J., Thomalla F. (2003). Resilience to natural hazards: How useful is this concept?. Glob. Environ. Chang. Part B Environ. Hazards.

[123] Kalisch R., Müller M.B., Tüscher O. (2015). A conceptual framework for the neurobiological study of resilience. Behav. Brain Sci..

[124] Windle G. (2011). What is resilience? A review and concept analysis. Rev. Clin. Gerontol..

[125] Xing C., Sun J.M. (2013). The role of psychological resilience and positive affect in risky decision-making. Int. J. Psychol..

[126] Lazarus R.S. (1993). From psychological stress to the emotions: A history of changing outlooks. Annu. Rev. Psychol..

[127] Brooks R., Brooks R., Goldstein S. (2005). The power of parenting. Handbook of Resilience in Children.

[128] Tedeschi R Kilmer R. (2005). Assessing strengths, resilience, and growth to guide clinical interventions. Prof. Psychol. Res. Pract..

[129] Alvord M., Grados J. (2005). Enhancing resilience in children: A proactive approach. Prof. Psychol. Res. Pract..

[130] Brandão J.M., Mahfoud M., Gianordoli-Nascimento I.F. (2011). The construction of the concept of resilience in psychology: Discussing the origins of resilience. Paid (Ribeirão Preto).

[131] Yunes M.A.M. (2003). Psicologia positiva e resiliência: O foco no indivíduo e na família. Psicologia em Estudo Maringá.

[132] Gillespie B.M., Chaboyer W., Wallis M. (2007). Development of a theoretically derived model of resilience through concept analysis. Contemp. Nurse.

[133] Haase J.E., Peterson S.J., Peterson S.J., Bredow T.S. (2013). Resilience. Middle Range Theories: Application to Nursing Theories.

[134] Jacelon C.S. (1997). The trait and process of resilience. J. Adv. Nurs..

[135] Richardson G.E. (2002). The metatheory of resilience and resiliency. J. Clin. Psychol..

[136] Lemyre L., Clément M., Corneil W., Craig L., Boutette P., Tyshenko M., Karyakina N., Clarke R., Krewski D., GAP-Santé Research Team (2005). A psychosocial risk assessment and management framework to enhance response to CBRN terrorism threats and attacks. Biosecur. Bioterror. Biodef. Strategy Pract. Sci..

[137] Masten A.S., Narayan A.J. (2012). Child development, disaster, and war. Annu. Rev. Psychol..

[138] Norris F.H., Stevens S.P., Pfefferbaum B., Wyche K.F., Pfefferbaum R.L. (2008). Community resilience as a metaphor, theory, set of capacities, and strategy for disaster readiness. Am. J. Community Psychol..

[139] Levine K.A. (2009). Against all odds: Resilience in single mothers of children with disabilities. Soc. Work Health Care.

[140] Patterson J. (2002). Understanding family resilience. J. Clin. Psychol..

[141] Rutter M. (2012). Resilience: Causal pathways and social ecology. The Social Ecology of Resilience.

[142] Tusaie K., Puskar K., Sereika S.M. (2007). A predictive and moderating model of psychosocial resilience in adolescents. J. Nurs. Scholarsh..

[143] Hernandez P., Gangsei D., Engstrom D. (2007). Vicarious resilience: A new concept in work with those who survive trauma. Fam. Process..

[144] Lee I., Lee E.O., Kim H.S., Park Y.S., Song M., Park Y.H. (2004). Concept development of family resilience: A study of Korean families with a chronically ill child. J. Clin. Nurs..

[145] Woodgate R.L. (1999). A review of the literature on resilience in the adolescent with cancer: Part, II. J. Pediatr. Oncol. Nurs..

[146] Masten A.S. (2007). Resilience in developing systems: Progress and promise as the fourth wave rises. Dev. Psychopathol..

[147] Olson C.A., Bond L., Burns J.M., Vella-Brodrick D.A., Sawyer S.M. (2003). Adolescent resilience: A conceptual analysis. J. Adolesc..

[148] Takahashi K., Kato A., Igari T., Sase E., Shibanuma A., Kikuchi K., Nanishi K., Jimba M., Yasuoka J. (2015). Sense of coherence as a key to improve homebound status among older adults with urinary incontinence. Geriatr. Gerontol. Int..

[149] De Alfieri W., Costanzo S., Borgogni T. (2011). Biological resilience of older adults versus frailty. Med. Hypotheses.

[150] Paletti R. (2007). Recovery in context: Bereavement, culture, and the transformation of the therapeutic self. Death Stud..

[151] Benard B. (1995). Fostering resilience in children. ERIC Digest.

[152] Luthar S.S. Resilience: A construct of value?. Presented at the 104th Annual Convention of the American Psychological Association.

[153] Pangallo A., Zibarras L., Lewis R., Flaxman P. (2014). Resilience through the lens of interactionism: A systematic review. Psychol. Assess..

[154] Masten A.S., Hubbard J., Gest S., Tellegen A., Garmezy N., Ramirez M. (1999). Competence in the context of adversity: Pathways to resilience and maladaptation from childhood to late adolescence. Dev. Psychopathol..

[155] Werner E. (1993). Risk, resilience, and recovery: Perspectives from the Kauai Longitudinal Study. Dev. Psychopathol..

[156] Ungar M. (2011). The social ecology of resilience: Addressing contextual and cultural ambiguity of a nascent construct. Am. J. Orthopsychiatr..

[157] Dale S.K., Grimes T., Miller L., Ursillo A., Drainoni M.L. (2017). “In our stories”: The perspectives of women living with HIV on an evidence-based group intervention. J. Health Psychol..

[158] De Santis J.P., Florom-Smith A., Vermeesch A., Barroso S., DeLeon D.A. (2013). Motivation, management, and mastery: A theory of resilience in the context of HIV infection. J. Am. Psychiatr. Nurses Assoc..

[159] Kent M., Davis M.C., Stark S.L., Stewart L.A. (2011). A resilience-oriented treatment for posttraumatic stress disorder: Results of a preliminary randomized clinical trial. J. Trauma. Stress.

[160] Steinhardt M., Dolbier C. (2008). Evaluation of a resilience intervention to enhance coping strategies and protective factors and decrease symptomatology. J. Am. Coll. Health.

[161] Bekhet A.K. (2013). Effects of positive cognitions and resourcefulness on caregiver burden among caregivers of persons with dementia. Int. J. Mental Health Nurs..

[162] Fernández-Lansac V., Crespo L.M., Cáceres R., Rodríguez-Poyo M. (2012). Resilience in caregivers of patients with dementia: A preliminary study. Rev. Esp. De Geriatr. Y Gerontol..

[163] O’Rourke N., Kupferschmidt A.L., Claxton A., Smith J.Z., Chappell N., Beattie B.L. (2010). Psychological resilience predicts depressive symptoms among spouses of persons with Alzheimer disease over time. Aging Ment. Health.

[164] Bull M.J. (2014). Strategies for sustaining self used by family caregivers for older adults with dementia. J. Holist. Nurs..

[165] Fitzpatrick K.E., Vacha-Haase T. (2012). Marital satisfaction and resilience in caregivers of spouses with dementia. Clin. Gerontol..

[166] Garces S.B.B., De Rosso Krug M., Hansen Brunelli A.V., da Costa F.T.L., Rosa C.B., Seibel R. (2012). Avaliação da resiliência do cuidador de idosos com Alzheimer. Revista Brasileira de Geriatria e Gerontologia.

[167] Wilks S.E., Croom B. (2008). Perceived stress and resilience in Alzheimer’s disease caregivers: Testing moderation and mediation models of social support. Aging Ment. Health.

[168] Hilliard M.E., Harris M.A., Weissberg-Benchell J. (2012). Diabetes resilience: A model of risk and protection in type 1 diabetes. Curr. Diabetes Rep..

[169] Hofer M.A. (2006). Evolutionary basis of adaptation in resilience and vulnerability: Response to Cicchetti and Blender. Ann. N. Y. Acad. Sci..

[170] Schneiderman N., Ironson G., Siegel S.D. (2005). Stress and health: Psychological, behavioral, and biological determinants. Annu. Rev. Clin. Psychol..

[171] Lee T.Y., Cheung C.K., Kwong W.M. (2012). Resilience as a positive youth development construct: A conceptual review. Sci. World J..

[172] Masten A., Best K., Garmezy N. (1990). Resilience and development: Contributions from the study of children who overcome adversity. Dev. Psychopathol..

[173] Thomas L.J., Revell S.H. (2016). Resilience in nursing students: An integrative review. Nurse Educ. Today.

[174] McAllister M., McKinnon J. (2009). The importance of teaching and learning resilience in the health disciplines: A critical review of the literature. Nurse Educ. Today.

[175] Thompson K.A., Bulls H.W., Sibille K.T., Bartley E.J., Glover T.L., Terry E.L., Vaughn I.A., Cardoso J.S., Sotolongo A., Staud R. (2018). Optimism and psychological resilience are beneficially associated with measures of clinical and experimental pain in adults with or at risk for knee osteoarthritis. Clin. J. Pain.

[176] Norman E. (2012). Resiliency Enhancement: Putting the Strength Perspective into Social Work Practice.

[177] Rabkin J.G., Remien R., Katoff L. (1993). Williams JBW. Resilience in adversity among long-term survivors of AIDS. Hosp. Community Psychiatry.

[178] Hart P., Brannan J., Chesma M. (2014). Resilience in nurses: An integrative review. J. Nurs. Manag..

[179] Eisenach J.H., Sprung J., Clark M.M., Shanafelt T.D., Johnson B.D., Kruse T.N., Long T.R. (2014). The Psychological and Physiological Effects of Acute Occupational Stress in New Anesthesiology Residents. A Pilot Trial. J. Am. Soc. Anesthesiol..

[180] Vaishnavi S., Connor K., Davidson J.R. (2007). An abbreviated version of the Connor-Davidson Resilience Scale (CD-RISC), the CD-RISC2: Psychometric properties and applications in psychopharmacological trials. Psychiatry Res..

[181] Catalano R.F., Berglund M.L., Ryan J.A.M., Lonczak H.S., Hawkins J.D. (2004). Positive youth development in the United States: Research findings on evaluations of positive youth development programs. Ann. Am. Acad. Political Soc. Sci..

[182] Wagnild G.M., Collins J.A. (2009). Assessing resilience. J. Psychosoc. Nurs. Ment. Health Serv..

[183] Wang L., Shi Z., Zhang Y., Zhang Z. (2010). Psychometric properties of the 10-item Connor–Davidson Resilience Scale in Chinese earthquake victims. Psychiatry Clin. Neurosci..

[184] Miller A.M., Chandler P.J. (2002). Acculturation, resilience, and depression in midlife women from the former Soviet Union. Nurs. Res..

[185] Wagnild G.M.H.M. (1993). Development and psychometric evaluation of the Resilience Scale. J. Nurs. Meas..

[186] Gibson C.A. (2010). An integrated approach to managing disruption-related risk: Life and death in a model community. J. Bus. Contin. Emerg. Plan..

[187] Horn S.R., Charney D.S., Feder A. (2016). Understanding resilience: New approaches for preventing and treating PTSD. Exp. Neurol..

[188] Cosco T.D., Kaushal A., Hardy R., Richards M., Kuh D., Stafford M. (2017). Operationalising resilience in longitudinal studies: A systematic review of methodological approaches. J. Epidemiol. Community Health.

[189] Luthar S.S., Doernberger C.H., Zigler E. (1993). Resilience is not a unidimensional construct: Insights from a prospective study of inner-city adolescents. Dev. Psychopathol..

[190] Masten A.S., Coatsworth J.D. (1998). The development of competence in favourable and unfavourable environments: Lessons from research on successful children. Am. Psychol..

[191] Waugh C.E., Fredrickson B.L., Taylor S.F. (2008). Adapting to life’s slings and arrows: Individual differences in resilience when recovering from an anticipated threat. J. Res. Personal..

[192] Masten A.S. (2015). Pathways to integrated resilience science. Psychol. Inq. Int. J. Adv. Psychol. Theory.

[193] Earnshaw V.A., Bogart L.M., Dovidio J.F., Williams D.R. (2013). Stigma and racial/ethnic HIV disparities: Moving toward resilience. Am. Psychol..

[194] Unger M. (2008). Resilience across cultures. Br. J. Soc. Work.

